# The Effect of Interdialytic Combined Resistance and Aerobic Exercise Training on Health Related Outcomes in Chronic Hemodialysis Patients: The Tunisian Randomized Controlled Study

**DOI:** 10.3389/fphys.2017.00288

**Published:** 2017-05-31

**Authors:** Bechir Frih, Hamdi Jaafar, Wajdi Mkacher, Zohra Ben Salah, Mohamed Hammami, Ameur Frih

**Affiliations:** ^1^Department of Biochemistry, Human Nutrition and Metabolic Disorders, Functional Foods and Vascular Health (LR12ES05), Faculty of Medicine of Monastir, University of MonastirMonastir, Tunisia; ^2^Institut du Savoir Montfort—Recherche, Hôpital MontfortOttawa, ON, Canada; ^3^Biochemistry, Microbiology and Immunology Department, Faculty of Medicine, University of OttawaOttawa, ON, Canada; ^4^Department of Research, Exercise Physiology and Pathophysiology, Biology, Medicine and Health (UR12ES06), Faculty of Medicine of Sousse, University of MonastirSousse, Tunisia; ^5^Department of Physical Medicine and Rehabilitation, CHU Fatuma Bourguiba MonastirMonastir, Tunisia; ^6^Nephrology and Internal Medicine Service, CHU Fatuma Bourguiba MonastirMonastir, Tunisia

**Keywords:** exercise, physical capacity, hemodialysis, physiological responses, psychological responses

## Abstract

**Background:** Tunisia has the highest prevalence of hemodialysis patients compared to the other countries in North Africa. Dialysis centers rarely offer an exercise program to prevent physiological and psychological dialysis therapy-related alterations in chronic hemodialysis patients.

**Aim:** To examine the effect of combined endurance-resistance training program on physiological and psychological outcomes in patients undergoing hemodialysis.

**Methods:** We designed a single blinded, randomized, controlled study for a period of 4 months. Patients were randomized to intervention group or control group. Intervention group patients received 4 training sessions per week, held on non-hemodialysis days for a period of 4 months, whereas control group patients continued their regular lifestyle practice without direct intervention from the personnel of this investigation. Patients were evaluated at baseline (initial assessment) and after the four-month study period (final assessment) by the same investigator blinded to treatment group assignment using physical, physiological, and psychological measurements.

**Results:** Compared with control group, intervention group showed significant improvement in physical performance during the sit-to-stand-to-sit tests (STS-10: −16.2%, ES = −1.65; STS-60: +23.43%, ES = 1.18), handgrip force task (+23.54%, ES = 1.16), timed up and go test (−13.86%, ES = −1.13), and 6-min walk test (+15.94%, ES = 2.09). Likewise, mini nutritional assessment long form scores after intervention period were significantly higher in the intervention group compared to the control group (ES = 1.43). Physical and mental component scores of SF-36 questionnaire increased significantly in the intervention group (ES = 1.10 and ES = 2.06, respectively), whereas hospital anxiety and depression scale scores decreased significantly (ES = −1.65 and ES = −2.72, respectively). Regarding biological parameters, intervention group displayed improvement in systolic and diastolic blood pressures (ES = −2.77 and ES = −0.87, respectively), HDL-cholesterol, LDL-cholesterol, and triglycerides systematic levels (ES = 1.15, ES = −0.98, and ES = −1.01, respectively); however no significant effect of intervention period was observed on C-reactive protein, hemoglobin, albumin, and total cholesterol levels (*P* > 0.05).

**Conclusion:** The current study showed that combined endurance-resistance training program had a beneficial effect on physical capacity and quality of life in chronic hemodialysis patients.

## Introduction

Chronic kidney disease is characterized by a progressive loss of renal function and considered as a serious public health problem worldwide with incidence much higher in the developing world. In North Africa, the reported annual incidence of end-stage kidney disease ranges from 34 to 200 cases per million population, depending on the country (Barsoum, 2003; Counil et al., 2008). Hemodialysis (HD) remains the most frequent renal replacement therapy for patients at end-stage kidney disease particularly in developing countries. In North Africa, Tunisia has the highest prevalence of HD patients with 680 patients per million population compared with the other countries (Benghanem Gharbi, 2010).

It is well-known that deconditioning infers the loss of physical capacity in response to physical inactivity or chronic disease and refers to a decreased aerobic fitness, muscle force, and endurance (Verbunt et al., 2005). The deconditioning spiral associated with end-stage renal disease (Painter, 1994) arises from inactivity which has a major impact on normal physical function and will result in disability associated with an increased mortality risk in people undergoing HD (Moore et al., 1993; Johansen et al., 2003; Sietsema et al., 2004). Indeed, these patients have a reduced aerobic fitness with a decreased peak oxygen uptake and skeletal muscle wasting. This wasting occurs due to factors such as aging, dietary intake, sedentary behavior, and comorbid illnesses (Clyne, 1996; Pupim et al., 2004). Dialysis treatment also induces significant metabolic changes such as hypovolemia due to ultrafiltration, rapid changes in electrolyte concentrations, and systemic inflammation, all of which can adversely affect physical function (Cheema et al., 2005).

Previous reports showed that HD patients have impaired mobility and balance (Blake and O'Meara, 2004), which is linked to an elevated risk of falling (Abdel-Rahman et al., 2011; Rossier et al., 2012). This can often result in multiple complications such as injuries, disability, loss of independence, poor quality of life, and nursing home placement (Cook and Jassal, 2005). Moreover, these complications yield high cost to the health system particularly in developing countries, e.g., dialysis expenditures in Tunisia represent as much as 4.5% of the health budget compared with 2.5% in France (Counil et al., 2008). The attempt to change sedentary behavior in HD population is relatively recent in Tunisia, where the dialysis centers rarely offer an exercise program in primary prevention strategies for HD-associated complications.

Numerous health-related benefits are derived from engaging in various physical training regimens (e.g., endurance or resistance training) in frail elderly persons and patients with a wide range of chronic diseases (Singh, 2002). In patients undergoing HD, most of previous studies examined the effects of intradialytic (i.e., during dialysis treatment) endurance or resistance training on physiological outcomes (Anderson et al., 2004; Pupim et al., 2004; Parsons et al., 2006). To our knowledge, no studies have examined the effect of interdialytic (i.e., on non-hemodialysis days) combined endurance-resistance training program on both physiological and psychological outcomes in HD patients using the magnitude-based inferences as proposed by Hopkins et al. (2009). In the current study, we compared changes in outcome variables to the smallest worthwhile change (SWC). We also reported both quantitatively and qualitatively the probabilities for these changes to be real for each measured variable. Hence, the aim of this study was to examine the effects of an adapted endurance-resistance training program held on non-hemodialysis days on physical capacity, biochemical markers, nutritional status, and psychological outcomes in Tunisian chronic HD patients. We hypothesized that interdialytic combined endurance-resistance exercise training would improve health-related outcomes.

## Materials and methods

### Participants

**Figure 1:**
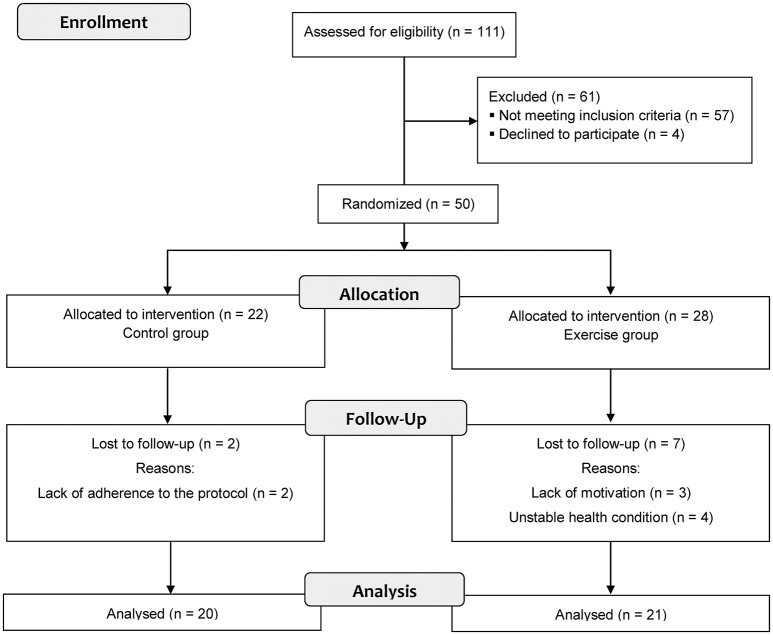
Flow diagram of the progress through the study phases.

We recruited patients undergoing HD from the Nephrology and Internal Medicine Service of CHU Monastir in Tunisia. The study was conducted between March 2012 and June 2013. Participation in the study was voluntary. Exclusion criteria were as follows: (i) patients who had chronic lung disease, (ii) patients who had ischemic heart disease, (iii) patients who had uncontrolled arrhythmias or hypertension, (iv) patients who had hemodynamic instability or musculoskeletal disorders exacerbated by exercise, and (v) patients who had been exercising regularly before starting the experiment. Eligible patients were randomized into two groups using a computer-generated randomization list. A flow diagram shows the progress of the patients through the phases of the study (Figure 1). Intervention group consisted of 21 exercising male patients and control group consisted of 20 non-exercising male patients (Table 1). The study protocol was approved by the local Ethics Committee on Human Research (ECHR) of CHU Monastir (Tunisia). All experimental procedures were carried out in accordance with the principles outlined in the Declaration of Helsinki. Prior to any data collection, all patients were fully informed about experimental procedures and were asked to adhere to the experimental protocol. Each patient provided written informed consent.

**Table 1:** Baseline characteristics for the two groups.

**Characteristic**	**Control group (*n* = 20)**	**Intervention group (*n* = 21)**
Age (years)	65.2 ± 3.1	64.2 ± 3.4
Dialysis prescription (h, days)	4 h, 3 days	4 h, 3 days
Months on hemodialysis	73.6 ± 13.4	72.7 ± 12.7
BMI (kg/m^2^)	24.3 ± 3.2	25.4 ± 2.8

### Study design

We designed a single blinded, randomized, controlled trial to examine the effect of endurance-resistance training program on physical capacity, biochemical markers, nutritional status, and psychological outcomes in chronic HD patients. Control group patients continued their regular lifestyle practice without direct intervention from the personnel of this investigation but underwent the same testing battery and participated in all assessments. Intervention group patients received 4 training sessions per week for a period of 4 months. All patients underwent 3 testing sessions before intervention (initial assessment) and 3 identical testing sessions immediately after the four-month intervention period (final assessment). The experimental sessions were conducted on non-dialysis days, 18–24 h following a dialysis treatment. On the first visit, patients completed the medical outcomes study short form 36-item (SF-36) and hospital anxiety and depression scale (HADS) questionnaires and underwent 24-h ambulatory blood pressure monitoring (ABPM). Blood samples (15 mL) were also collected in EDTA-containing tubes and immediately centrifuged (4°C) for 15 min at 3,000 rpm. Serum was stored at −80°C until further analysis. On the second visit, patients were assessed for nutritional status using the mini nutritional assessment long form (MNA-LF) and carried out sit-to-stand-to-sit tests (STS). On the third visit, patients performed the handgrip task, the timed up and go test (TUG), and the 6-min walk test (6MWT). Physical tests were interspaced by a period of recovery. All assessments were performed by the same experienced investigator who was blinded as to the patients' group assignment.

### Training program

The training program had been previously developed with the Department of Physical Medicine and Rehabilitation of CHU Monastir, Tunisia (Frih et al., 2017). All training sessions (64 sessions) were supervised by two professional physiotherapy and physical training technicians. Training session was done four-fold weekly in non-hemodialysis days and lasted for ~60 min. The training program was composed of both resistance and endurance training exercises. Each training session began with a 10-min warm-up period progressing from isolated mobilizing to gross mobilizing exercises. Resistance program specifically targeted anaerobic capacity where patients performed dynamic closed and open-chain strengthening exercises. For this purpose, the quadriceps muscles, pectoral muscles, triceps brachia muscles, biceps brachia muscles, and hamstrings were trained on a multigym. Patients started at 50% of their initial one-repetition maximum (1-RM) with 12–15 repetitions for each exercise. Each month the load was increased by 5% of the 1-RM. Endurance program specifically targeted aerobic capacity where patients performed ergometer cycling and treadmill walking for 20 min. Exercise intensities were adapted to each patient by monitoring heart rate (Polar RS400, Polar Electro, Finland) and a Borg score of 5–6 for dyspnea or fatigue was set as a target (Borg, 1982). In order to avoid monotony, patients were encouraged to perform as many bouts as possible with the greatest variety of exercises during each training session. Additionally, a datasheet was created for each patient to monitor the appearance of adverse effects related to exercise, such as symptomatic hypotension and severe muscle symptoms (e.g., pain, muscle cramps). Each training session ended with a 10-min cool-down period consisting of stretching and balancing exercises.

### Biochemical analyses

Biochemical assays were performed using standard techniques at the Central Laboratory of the University Hospital, Monastir, Tunisia. Hemoglobin was analyzed by routine high-performance liquid chromatography based ion-exchange procedure (HA-8140, Rungis, France). Serum albumin was measured by the bromcresol green method. Total cholesterol, HDL-cholesterol, and triglyceride levels were determined using spectrophotometric methods (AU2700-Olympus, Beckman Coulter). LDL-cholesterol was then calculated using the Friedewald formula. C-reactive protein was measured by immunoturbidimetry (Dupuy et al., 2003). These parameters were measured in fasting blood samples.

### Ambulatory blood pressure monitoring (ABPM)

For blood pressure assessment, patients underwent 24-h ABPM during the non-dialysis days. ABPM was performed using oscillometric monitor (SpaceLabs 90207, Spacelabs Medical, Créteil, France). This device was programmed to take readings at 20-min intervals during the wakeful period (from 7:00 to 23:00 h) and at 30-min intervals during the sleeping period. During the monitoring, patients were instructed to maintain their normal activities.

### Medical outcomes study short form 36-item (SF-36)

The SF-36 questionnaire was used to evaluate self-reported domains of health status (Ware and Sherbourne, 1992). This questionnaire consists of 36 items compiled into 8 scales: physical functioning (PF), role functioning/physical (RP), bodily pain (BP), general health (GH), vitality (VT), social functioning (SF), role functioning/emotional (RE), and mental health (MH). These scales range from 0 to 100; a higher score is more positive (i.e., less pain or less limitation). Normalized scores representing overall physical functioning and mental functioning are calculated from the individual scales and are presented as the physical component scale (PCS) and the mental component scale (MCS). The PCS includes the following dimensions: PF, RP, BP, GH, VT, and SF. The MCS is composed by the RE and MH items, and includes elements of the GH, VT, and SF scales (Ware, 2000).

### Hospital anxiety and depression scale (HADS)

The HADS was developed to assess both anxiety and depression. This questionnaire consists of an anxiety symptoms subscale and a depressive symptoms subscale (Zigmond and Snaith, 1983). It has been found to be a valid assessment of the risk in primary care (Zigmond and Snaith, 1983).

### Sit-to-stand-to-sit tests (STS)

The STS-10 and STS-60 were performed to measure lower-extremity muscle capacity force (Csuka and McCarty, 1985). The STS-10 measures the time in s required to complete 10 consecutive repetitions of sitting down on and getting up from a chair, whereas the STS-60 measures the number of repetitions achieved in 60 s. The STS-10 was performed first, and the STS-60 was performed after a 15-min recovery period. During the STS-10, patients were instructed to perform the task as fast as possible, starting and finishing at the sitting position. Patients were allowed a practice trial before the beginning of the test. They began the test by crossing their arms on their chest and sitting with their back against the chair (44 cm high and 38 cm deep), which was backed up against a wall in order to minimize the risk of falling (Cho et al., 2004; Whitney et al., 2005). This test is simple, inexpensive, rapid, and reproducible (Csuka and McCarty, 1985) and is included in the battery of tests used for people with renal disease (Headley et al., 2002). The STS-60 measured the number of repetitions of sitting down on and getting up from a chair achieved in 60 s. The number of repetitions achieved was recorded at the end of the test for analysis. The STS-60 has been found to be a valid measure of lower body muscle endurance force (Ritchie et al., 2005).

### Six-minute walk test (6MWT)

The 6MWT was conducted according to the recommendations of the American Thoracic Society (ATS, 2002). The 6MWT was performed in a 20-m-long corridor; tape was placed every 2 m. Patients were asked to walk the longest distance possible in 6 min by walking continuously the 20 m indicated on the floor, turning around at the final mark without stopping, and covering as much ground as possible (ATS, 2002). The standardized instruction given to the patients was as follows: Walk as far as possible for 6 min, but don't run or jog (ATS, 2002). They could stop if needed and restart later. The distance covered in meters was recorded at the end of the test for analysis. The 6MWT is a simple, valid tool for testing the ability to perform activities of daily life (e.g., walking; Fitts and Guthrie, 1995). The physiological exercise capacity testing usually requires people to perform at maximal or nearly maximal levels in laboratory-based environments and involves techniques and tasks with which people are not always familiar.

### Timed up and go test (TUG)

The TUG was used to provide a timed measure of balance and functional mobility (Podsiadlo and Richardson, 1991). The test requires the patient to rise from a standard armchair, walk 3 m at a comfortable pace, walk back to the chair, and sit down. A practice trial was performed. Normative data are available for elderly individuals who reside in the community (Steffen et al., 2002). The TUG has high intra- and inter-reliability and predictive validity for falls in community living adults (Podsiadlo and Richardson, 1991). A cut-off score of 16 s or more has been shown to predict falls in community-dwelling elderly (Okumiya et al., 1998).

### Handgrip force dynamometry

Handgrip force was measured using a dynamometer (T.K.K. 5401 GRIP D, Takei Science Instruments, Niigata, Japan). Patients were positioned standing with the elbow extended at the moment of the test, according to the manufacturer's instructions. Three consecutive repetitions of 4 s, interspaced by 90 s of rest, were performed with the dominant arm. Verbal encouragement was given during the task and the highest peak force was used for analysis.

### Nutritional status

Nutritional status was assessed using the mini nutritional assessment long form (MNA-LF) tool (Guigoz et al., 1996), which is an internationally validated method for the nutritional assessment of elderly patients (Vellas et al., 1999). The MNA-LF consists of a screening part and an assessment part. The screening part contains 6 items concerning decline of food intake, weight loss in the past 3 months, acute mobility, disease/distress, neuropsychological problems, and additional anthropometric measures. The assessment part consists of 12 items concerning housing, medicine use, pressure ulcers, dietary intake, and self-rated nutritional and health status. The MNA-LF provides a total score that ranges from 0 to 30 points. A score ≤ 17 points is regarded as evidence of malnutrition, 18–23 points indicate a risk of malnutrition, and ≥24 points an adequate nutritional status (Beck et al., 1999).

### Statistical analysis

The Statistica software version 10.0 for windows (StatSoft, Maisons-Alfort, France) was used for data analysis. Data are reported as mean and standard deviation (± SD). The distribution of each variable was examined using the Shapiro-Wilk test for normality. Data were analyzed using a mixed measures (group × period) analysis of variance (ANOVA) for repeated measures. Bonferroni tests were used as a means by which to locate significant differences. The level of significance was predetermined to be *P* < 0.05 for all statistical analyses. Relative changes (%) with corresponding 95% confidence interval (95% CI) were calculated for each physical performance variable. Additionally, data were assessed for clinical significance using an approach based on the magnitude of changes proposed by Hopkins et al. (2009). For between-group comparisons, the effect size (ES) of change with corresponding 95% confidence interval (95% CI) in each measured variable between the control and intervention groups was calculated using the pooled SD (Cohen, 1992). Threshold values for the interpretation of ES were 0–0.19 (trivial), 0.2–0.49 (small), 0.50–0.79 (medium), >0.79 (large). For between-group comparisons, the likelihood that the true values of the estimated difference were better/beneficial, similar, or worse/detrimental than the SWC was calculated for each variable (Hopkins et al., 2009). Threshold values for assigning quantitative chances of better/beneficial or poorer/detrimental effect were: <1%, almost certainly not; 1–5%, very unlikely; 5–25%, unlikely; 25–75%, possible; 75–95%, likely; 95–99%, very likely; and >99%, almost certain. The true difference was assessed as unclear when the chance of having better/beneficial or poorer/detrimental value was both >10% (Hopkins et al., 2009).

## Results

### Physical capacity

**Figure 2:**
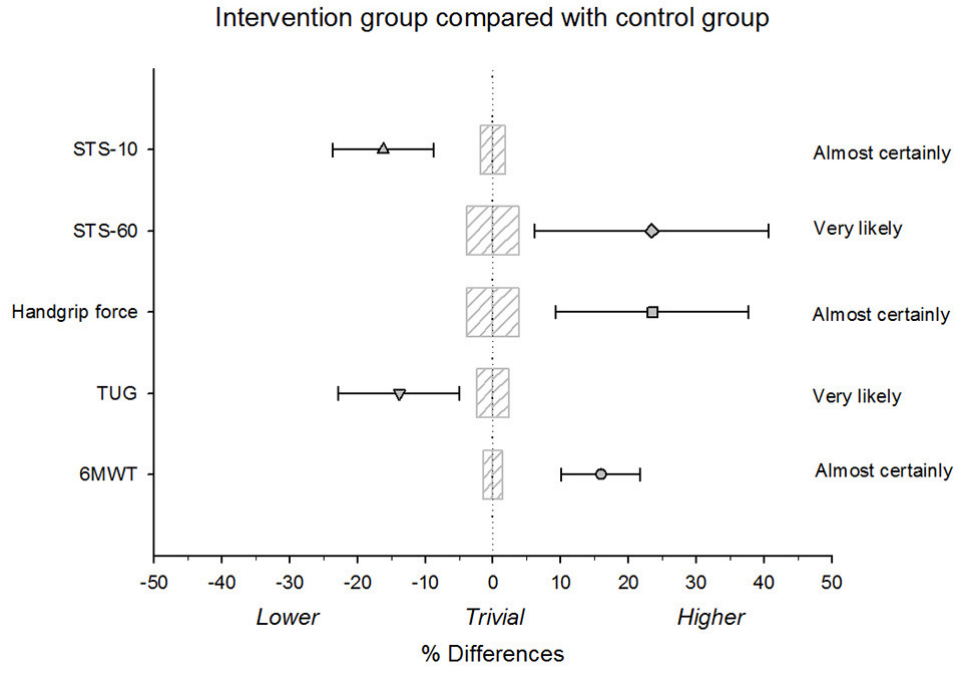
Relative differences between the intervention group and the control group for the 6-min walk test (6MWT), timed up and go test (TUG), handgrip force, and sit-to-stand-to-sit tests (STS-10 and STS-60). Bars indicate uncertainty in the true mean changes with 95% confidence intervals. Trivial areas were calculated based on the smallest worthwhile change (SWC).

The mean values of physical capacity tests are displayed in Table 2. The analysis revealed a significant group × period interaction effect for STS-10 [*F*_(1, 39)_ = 19.60, *P* < 0.001], STS-60 [*F*_(1, 39)_ = 7.76, *P* = 0.008], handgrip force [*F*_(1, 39)_ = 12.32, *P* = 0.001], TUG [*F*_(1, 39)_ = 9.71, *P* = 0.003], and 6MWT [*F*_(1, 39)_ = 32.59, *P* < 0.001]. *Post-hoc* test revealed a significant improvement in performance during the STS-10 (−16.2%), STS-60 (+23.43%), handgrip force (+23.54%), TUG (−13.86%), and 6MWT (+15.94%) tests for the intervention group compared to the control group (*P* < 0.001, Figure 2). Practically worthwhile differences between the control and intervention groups were also evident, as supported by large ESs and qualitative outcomes, suggesting very likely to almost certainly true changes (Table 2, Figure 2).

**Table 2:** Baseline and final assessments for physical capacity in both groups.

**Outcome variable**	**Control group (*****n*** = **20)**		**Intervention group (*****n*** = **21)**		**Changes observed for the intervention group compared to the control group**		
	**Initial assessment**	**Final assessment**	**Initial assessment**	**Final assessment**	**Effect size (95% CI)**	**Rating**	**Chances of beneficial/trivial/detrimental effect (%)**
STS-10 (s)	31.7 ± 2.5	32 ± 3.1	32 ± 3.5	27 ± 2.9^*^	−1.65 (−2.41 to −0.90)	Large	100/0/0
STS-60	21.6 ± 4.2	21.7 ± 4.1	21.5 ± 4.2	26.6 ± 3.5^*^	1.18 (0.31 to 2.05)	Large	99/1/0
Handgrip force (N)	29.3 ± 5.6	30 ± 5.2	29.8 ± 6.0	37.4 ± 4.8^*^	1.16 (0.46 to 1.86)	Large	100/0/0
TUG (s)	15.3 ± 1.6	15.2 ± 1.9	15.1 ± 2.0	12.9 ± 1.6^*^	−1.13 (−1.86 to −0.40)	Large	99/1/0
6MWT (m)	422.2 ± 26.6	415.6 ± 36.3	420 ± 35.1	480.5 ± 31.9^*^	2.09 (1.33 to 2.85)	Large	100/0/0

*STS-10, sit-to-stand-to-sit 10 test; STS-60, sit-to-stand-to-sit 60 test; TUG, timed up and go test; 6MWT, 6-min walk test*.

* *Significantly different from baseline (P < 0.05)*.

### Biological parameters

Table 3 shows the results of baseline and final assessments for both groups. Analysis showed a significant group × period interaction effect for systolic and diastolic blood pressures [*F*_(1, 39)_ = 66.87, *P* < 0.001 and *F*_(1, 39)_ = 11.38, *P* = 0.002, respectively]. *Post-hoc* analysis revealed that final assessments of these parameters were significantly lower compared to initial assessments in the intervention group. Likewise, large ESs were obtained for the differences between the intervention group and the control group with almost certainly and very likely beneficial effect of the training program (Table 3). A significant group × period interaction effect was obtained for HDL-cholesterol [*F*_(1, 39)_ = 4.91, *P* = 0.032], LDL-cholesterol [*F*_(1, 39)_ = 6.30, *P* = 0.016], and triglycerides levels [*F*_(1, 39)_ = 6.18, *P* = 0.017]. *Post-hoc* analysis revealed that HDL-cholesterol was significantly higher at the final assessment, whereas LDL-cholesterol and triglycerides were significantly lower at the final assessments compared to baseline values in the intervention group. Moreover, large ESs were observed for these parameters with qualitative outcomes suggesting likely beneficial effect of the training program (Table 3).

**Table 3:** Baseline and final assessments for biological parameters in both groups.

**Variable**	**Control group (*****n*** = **20)**		**Intervention group (*****n*** = **21)**		**Changes observed for the intervention group compared to the control group**		
	**Initial assessment**	**Final assessment**	**Initial assessment**	**Final assessment**	**Effect size (95% CI)**	**Rating**	**Chances of beneficial/trivial/detrimental effect (%)**
Systolic blood pressure (mmHg)	148 ± 6	149.2 ± 5.1	147.5 ± 4.1	134.1 ± 5.2^*^	−2.77 (−3.48 to −2.07)	Large	100/0/0
Diastolic blood pressure (mmHg)	80.5 ± 4.7	78.4 ± 3.4	78.8 ± 3.6	73 ± 3.6^*^	−0.87 (−1.40 to −0.34)	Large	99/1/0
C-reactive protein (mg/L)	4.1 ± 1.1	4 ± 1.4	4.1 ± 1.3	4.1 ± 1.2	0.09 (−0.76 to 0.94)	Trivial	40/36/25
Hemoglobin (g/dL)	10.1 ± 2	10 ± 1.6	10.2 ± 1.8	10.4 ± 1.7	0.17 (−0.58 to 0.91)	Trivial	47/37/16
Albumin (g/L)	39.9 ± 3.7	40.4 ± 3.7	39.6 ± 3.5	40 ± 2.6	−0.10 (−0.41 to 0.39)	Trivial	17/68/15
Total cholesterol (mmol/L)	4.2 ± 0.4	4.1 ± 0.6	4 ± 0.5	3.9 ± 0.4	−0.12 (1.10 to 0.87)	Trivial	43/31/26
HDL-cholesterol (mmol/L)	1.3 ± 0.4	1.4 ± 0.4	1.2 ± 0.3	1.7 ± 0.4^*^	1.15 (0.10 to 2.19)	Large	96/3/1
LDL-cholesterol (mmol/L)	2.6 ± 0.5	2.5 ± 0.4	2.4 ± 0.3	1.9 ± 0.3^*^	−0.98 (−1.79 to −0.16)	Large	97/3/0
Triglycerides (mmol/L)	1.9 ± 0.6	1.9 ± 0.5	1.9 ± 0.5	1.4 ± 0.4^*^	−1.01 (−1.83 to −0.19)	Large	98/2/0

* *Significantly different from baseline (P < 0.05)*.

No significant group × period interaction effect was observed for C-reactive protein [*F*_(1, 39)_ = 0.04, *P* = 0.834], hemoglobin [*F*_(1, 39)_ = 0.21, *P* = 0.648], albumin [*F*_(1, 39)_ = 0.001, *P* = 0.976], and total cholesterol levels [*F*_(1, 39)_ = 0.06, *P* = 0.806]. Likewise, trivial ESs were obtained for these variables with qualitative outcomes suggesting unclear effect (Table 3).

### Nutritional status

Table 4 presents MNA-LF scores for both groups. The analysis of MNA-LF scores revealed a significant group × period interaction effect [*F*_(1, 39)_ = 25.27, *P* < 0.001]. *Post-hoc* analysis revealed that MNA-LF scores after intervention period were significantly higher in the intervention group compared to the control group (ES = 1.43 [0.86 to 2.00], Rating: Large). Chances that the true differences were beneficial/trivial/detrimental effect were 100/0/0% (Almost certainly). Indeed, patients in the intervention group were 4.8% undernourished, 85.7% at risk of malnutrition, and 9.5% normal nourished at initial assessment. After intervention period, the percentages were 4.8% undernourished, 23.8% at risk of malnutrition, and 71.4% normal nourished (Table 4).

**Table 4:** Mini nutritional assessment long form (MNA-LF) scores at initial and final assessments in both groups.

	**Control group (*****n*** = **20)**		**Intervention group (*****n*** = **21)**	
	**Initial assessment**	**Final assessment**	**Initial assessment**	**Final assessment**
Scores	21.3 ± 2.3	21.8 ± 1.9	21.2 ± 2	24.9 ± 2.4
Undernourished	2 (10%)	1 (5%)	1 (4.8%)	1 (4.8%)
Risk of malnutrition	14 (70%)	17 (85%)	18 (85.7%)	5 (23.8%)
Normal nourished	4 (20%)	2 (10%)	2 (9.5%)	15 (71.4%)

### Psychological parameters

**Figure 3:**
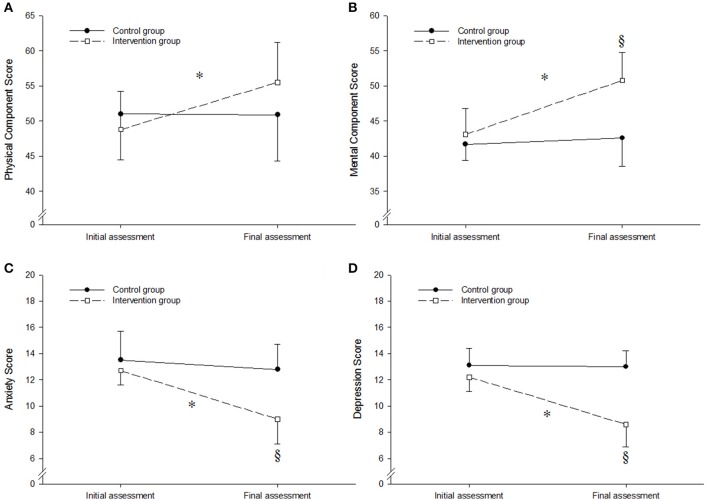
Physical component scores **(A)**, mental component scores **(B)**, anxiety scores **(C)** and depression scores **(D)** at initial and final assessments in both groups. ^*^Significantly different from initial assessment (*P* < 0.01). ^§^Significantly different from control group (*P* < 0.01). Values are means ± *SD*.

Figure 3 displays psychological scores at initial and final assessments for both groups. The analysis revealed a significant group × period interaction effect for physical and mental component scores of SF-36 [*F*_(1, 39)_ = 9.99, *P* = 0.003 and *F*_(1, 39)_ = 20.27, *P* < 0.001, respectively] as well as the anxiety and depression scores of HADS [*F*_(1, 39)_ = 16.07, *P* < 0.001 and *F*_(1, 39)_ = 43.91, *P* < 0.001, respectively]. *Post-hoc* tests revealed that physical and mental component scores increased significantly in the intervention group, whereas anxiety and depression scores decreased significantly. Differences between groups were also rated as large (ES ranged from 1.10 to 2.75) and qualitative outcome with almost certainly beneficial effect.

## Discussion

In the present investigation, we examined the effect of interdialytic endurance-resistance training on chronic HD patients' physiological and psychological outcomes. The main findings were that combined endurance-resistance training program has been shown to be very likely effective for enhancing physical capacity in chronic HD patients. Indeed, this training program lowered the total time in the STS-10 and TUG tests and improved handgrip force, the number of repetitions in the STS-60, and total distance covered during 6MWT. Likewise, it had a likely beneficial effect on blood pressure parameters, HDL-cholesterol, LDL-cholesterol, and triglycerides levels. This intervention modality impacts positively patients' nutritional status and psychological outcomes.

### Physical capacity

The physical tests used in the present study are widely used in clinical setting (ATS, 2002; Headley et al., 2002; Ritchie et al., 2005). In a reliability study, Segura-Orti and Martinez-Olmos (2011) reported high absolute reliability and lower relative test–retest reliability for the STS-10, STS-60, 6MWT, and handgrip force test in patients undergoing HD. In the present study, we assessed the effectiveness of the endurance-resistance exercise intervention in HD patients using controlled study design and an approach based on the magnitude of changes (Hopkins et al., 2009), which allows us to study quantitatively and qualitatively the observed changes for each performance variable.

The gold standard measure for assessment of physical function is the peak oxygen uptake in a graded exercise test. Because of the need for special and high-cost equipment and the limited functional status in HD patients, this test is less used in the clinical setting. Interestingly, the 6MWT is a useful tool and widely used to assess the effect of dialysis therapy on physical function (Segura-Orti and Martinez-Olmos, 2011). The mean 6MWT distance of our patients at the initial assessment was similar to values reported in previous studies conducted in HD patients (Painter et al., 2000; Headley et al., 2002; Parsons et al., 2006; Reboredo Mde et al., 2010). After intervention period, the 6MWT distance was significantly increased in the intervention group compared with the control group, indicating that the endurance-resistance program increased physical functioning in HD patients. These findings are in agreement with previous reports on exercise in HD patients (Parsons et al., 2006; Reboredo Mde et al., 2010). For instance, Reboredo Mde et al. (2010) showed that 3 months of aerobic exercise training performed during hemodialysis sessions increased significantly by 9% the 6MWT distance in 14 HD patients. In another study, Parsons et al. (2006) showed that 5 months of endurance exercise training during dialysis in 13 HD patients were associated with an increase of 14% in the 6MWT distance.

In the previous studies, the authors administered intradialytic exercise training program (Painter et al., 2000; Headley et al., 2002; Parsons et al., 2006; Reboredo Mde et al., 2010). However, performing physical exercise during HD may induce complications. Indeed, it is documented that dialysis therapy induces acute complications such as hypotension, muscular cramps, arrhythmia, nausea, vomiting, and headaches, among others, which could be magnified by exercising (Himmelfarb, 2005). In the current study, we used an interdialytic prescription, which showed better compliance with exercise training in HD patients. Additionally, this prescription allows various movements with wide range of motion that are not possible during dialysis.

The present study's results showed that the intervention program improves significantly force production of lower and upper body limbs in HD patients assessed by STS and handgrip force tests, respectively. The literature revealed that skeletal muscle wasting is common in HD patients, which is a strong predictor of mortality in this population (Carrero et al., 2008). This wasting contributes to reductions in muscular force and associated functional impairment such as impaired mobility and balance. In accordance with previous studies, resistance training may be efficient in terms of clinical outcomes in these patients (Cheema et al., 2005; Chan et al., 2007; Chen et al., 2010).

Previous reports showed that falls can be markers of poor health and declining function, and are often associated with significant morbidity and mortality in HD population (Li et al., 2008). It has been shown that a cut-off score of 16 s or more during the TUG predicts falls in older subjects (Okumiya et al., 1998). At the initial assessment of the current study, patients classified as at risk of falls were 30 and 35% for control and intervention group, respectively, whereas at final assessment these percentages were 40 and 0% for control and intervention group, respectively. These findings are in agreement with a recent study which reported a significant improvement in balance following a resistance-endurance training program in HD patients (Frih et al., 2017). Therefore, endurance-resistance exercise should be recommended as a central component of the exercise prescription for HD patients.

### Biological parameters

Regarding blood pressure parameters, the current study's results showed that both systolic and diastolic blood pressure levels of the intervention group decreased significantly after the intervention period. These results were in accordance with previous reports (Anderson et al., 2004; Reboredo Mde et al., 2010), which reinforces the benefit of exercise training for blood pressure control in HD patients.

Many patients with renal failure show abnormalities of lipid metabolism. Indeed, hypertriglyceridemia and low levels of HDL-cholesterol are frequent abnormalities in HD (Pennell et al., 2006). The various disturbances of lipoprotein metabolism in uremia can be summarized as decreased catabolism of lipoproteins with an inappropriate synthesis of LDL-cholesterol (Heuck and Ritz, 1980). There is a decreased lipoprotein catabolism, resulting in incompletely cleared intermediate particles and diminished formation of HDL-cholesterol. In the current study, no significant changes were observed for total cholesterol levels between groups, however, the intervention group increased HDL-cholesterol and reduced triglycerides and LDL-cholesterol levels after the training period. Therefore, exercise prescription may be useful to mitigate dyslipidemia, which is considered as a risk factor for cardiovascular disease in HD patients (Pennell et al., 2006).

Regarding systemic inflammation, C-reactive protein level was similar between the two groups after the intervention period. These findings are in agreement with the results reported by Wilund et al. (2010) after 4 months of intradialytic endurance exercise training. Likewise, Cheema et al. (2011) reported no significant improvement of circulating pro- and anti-inflammatory markers after 12 weeks of intradialytic progressive resistance training. In the present study, we did not measure circulating levels of other pro-inflammatory mediators such as tumor necrosis factor α and interleukin-6. Indeed, C-reactive protein is the major acute phase protein secreted by the liver due to interleukin-6 stimulation in humans (Heinrich et al., 1990). In another study, Cheema et al. (2007) showed that C-reactive protein level tended to decrease (*P* = 0.05) after prolonged intradialytic progressive resistance training (24 weeks) compared to short-duration training (12 weeks). Future studies with large sample and/or longer duration are required to determine the effects of exercise intervention on pro-inflammatory markers as well as anti-inflammatory cytokines in HD patients.

### Nutritional status

The literature revealed a causal relationship between malnutrition and poor physical performance particularly in elderly persons and patients (Vellas et al., 1999; Rossier et al., 2012). In the current study, improvement in functional capacity assessed by the 6MWT was associated with improvement in nutritional status assessed by MNA-LF (*r* = 0.51, *P* = 0.018). At initial assessment, normal nourished HD patients were 20 and 9.5% in the control group and intervention group, respectively, whereas after the intervention period 10 and 71.4% of patients in the control and intervention group were with adequate nutritional status, respectively. Furthermore, few exercise intervention studies have examined changes in dietary intake in HD patients. Therefore, studies aiming to examine changes in subjective ratings of appetite or possible changes in the composition of energy intakes (e.g., carbohydrate vs. fat vs. protein) are warranted.

### Psychological outcomes

Anxiety and depression disorders have been found to be associated with an increased risk of mortality and poor health-related quality of life in HD population (McKercher et al., 2013). Indeed, patients undergoing HD have low quality of life scores that are frequently related to hospitalization and death (Lowrie et al., 2003). Recent research studies suggested the role of physical activity in improving quality of life in various disorders such as patients with stroke, Parkinson's disease, and chronic low back pain (Moussouli et al., 2014; Rafferty et al., 2017). The present study's results revealed a beneficial effect of the training program on exercising patients' psychological outcomes. Indeed, the improvement in physical capacity was accompanied by a significant improvement in quality of life and decrease in anxiety and depression scores. These findings are in agreement with the results reported by other authors among HD patients after exercise training (Painter et al., 2000, 2002; van Vilsteren et al., 2005). Therefore, exercise training can be a safe supplement and non-pharmaceutical therapeutic agent for HD patients to reduce the severity of complications that occur during dialysis as well as to avoid the development of depression comorbidity.

### Limitations

Limitations of our study include the single geographical HD center utilized and lack of female patients due to cultural reasons. Furthermore, the four-month intervention period may have been too short to improve serum parameters such as markers of inflammation.

## Conclusion

The current study showed that combined endurance-resistance training program had a very likely beneficial effect on physiological and psychological outcomes in chronic HD patients. We recommend that nephrologists consider and implement such training programs as standard clinical practice in HD units.

## Author contributions

BF, HJ, WM, ZB, MH, and AF: design of the work, acquisition, analysis, and interpretation of data, drafting the work, final approval of work, and its integrity.

### Conflict of interest statement

The authors declare that the research was conducted in the absence of any commercial or financial relationships that could be construed as a potential conflict of interest. The reviewers, AAM and YS, and handling Editor declared their shared affiliation, and the handling Editor states that the process nevertheless met the standards of a fair and objective review.

## References

[B1] Abdel-Rahman E. M. Yan G. Turgut F. Balogun R. A. (2011). Long-term morbidity and mortality related to falls in hemodialysis patients: role of age and gender - a pilot study. Nephron Clin. Pract. 118, c278–c284. 10.1159/000322275 21212691

[B2] Anderson J. E. Boivin M. R. Jr. Hatchett L. (2004). Effect of exercise training on interdialytic ambulatory and treatment-related blood pressure in hemodialysis patients. Ren. Fail. 26, 539–544. 10.1081/JDI-200031735 15526912

[B3] ATS (2002). ATS statement: guidelines for the six-minute walk test. Am. J. Respir. Crit. Care Med. 166, 111–117. 10.1164/ajrccm.166.1.at1102 12091180

[B4] Barsoum R. S. (2003). End-stage renal disease in North Africa. Kidney Int. Suppl. 83, S111–S114. 10.1046/j.1523-1755.63.s83.23.x 12864887

[B5] Beck A. M. Ovesen L. Osler M. (1999). The ‘Mini Nutritional Assessment’ (MNA) and the ‘Determine Your Nutritional Health’ Checklist (NSI Checklist) as predictors of morbidity and mortality in an elderly Danish population. Br. J. Nutr. 81, 31–36. 10.1017/S0007114599000112 10341673

[B6] Benghanem Gharbi M. (2010). Renal replacement therapies for end-stage renal disease in North Africa. Clin. Nephrol. 74(Suppl. 1), S17–S19. 10.5414/CNP74S017 20979957

[B7] Blake C. O'Meara Y. M. (2004). Subjective and objective physical limitations in high-functioning renal dialysis patients. Nephrol. Dial. Transplant. 19, 3124–3129. 10.1093/ndt/gfh538 15494354

[B8] Borg G. A. (1982). Psychophysical bases of perceived exertion. Med. Sci. Sports Exerc. 14, 377–381. 10.1249/00005768-198205000-00012 7154893

[B9] Carrero J. J. Chmielewski M. Axelsson J. Snaedal S. Heimburger O. Barany P.. (2008). Muscle atrophy, inflammation and clinical outcome in incident and prevalent dialysis patients. Clin. Nutr. 27, 557–564. 10.1016/j.clnu.2008.04.007 18538898

[B10] Chan M. Cheema B. S. Fiatarone Singh M. A. (2007). Progressive resistance training and nutrition in renal failure. J. Ren. Nutr. 17, 84–87. 10.1053/j.jrn.2006.10.014 17198940

[B11] Cheema B. Abas H. Smith B. O'Sullivan A. Chan M. Patwardhan A.. (2007). Randomized controlled trial of intradialytic resistance training to target muscle wasting in ESRD: the Progressive Exercise for Anabolism in Kidney Disease (PEAK) study. Am. J. Kidney Dis. 50, 574–584. 10.1053/j.ajkd.2007.07.005 17900457

[B12] Cheema B. S. Abas H. Smith B. C. O'Sullivan A. J. Chan M. Patwardhan A.. (2011). Effect of resistance training during hemodialysis on circulating cytokines: a randomized controlled trial. Eur. J. Appl. Physiol. 111, 1437–1445. 10.1007/s00421-010-1763-5 21161265

[B13] Cheema B. S. Smith B. C. Singh M. A. (2005). A rationale for intradialytic exercise training as standard clinical practice in ESRD. Am. J. Kidney Dis. 45, 912–916. 10.1053/j.ajkd.2005.01.030 15861357

[B14] Chen J. L. Godfrey S. Ng T. T. Moorthi R. Liangos O. Ruthazer R.. (2010). Effect of intra-dialytic, low-intensity strength training on functional capacity in adult haemodialysis patients: a randomized pilot trial. Nephrol. Dial. Transplant. 25, 1936–1943. 10.1093/ndt/gfp739 20100734 PMC2902890

[B15] Cho B. L. Scarpace D. Alexander N. B. (2004). Tests of stepping as indicators of mobility, balance, and fall risk in balance-impaired older adults. J. Am. Geriatr. Soc. 52, 1168–1173. 10.1111/j.1532-5415.2004.52317.x 15209657

[B16] Clyne N. (1996). Physical working capacity in uremic patients. Scand. J. Urol. Nephrol. 30, 247–252. 10.3109/00365599609182300 8908642

[B17] Cohen J. (1992). A power primer. Psychol. Bull. 112, 155–159. 10.1037/0033-2909.112.1.155 19565683

[B18] Cook W. L. Jassal S. V. (2005). Prevalence of falls among seniors maintained on hemodialysis. Int. Urol. Nephrol. 37, 649–652. 10.1007/s11255-005-0396-9 16307356

[B19] Counil E. Cherni N. Kharrat M. Achour A. Trimech H. (2008). Trends of incident dialysis patients in Tunisia between 1992 and 2001. Am. J. Kidney Dis. 51, 463–470. 10.1053/j.ajkd.2007.10.032 18295062

[B20] Csuka M. McCarty D. J. (1985). Simple method for measurement of lower extremity muscle strength. Am. J. Med. 78, 77–81. 10.1016/0002-9343(85)90465-6 3966492

[B21] Dupuy A. M. Badiou S. Descomps B. Cristol J. P. (2003). Immunoturbidimetric determination of C-reactive protein (CRP) and high-sensitivity CRP on heparin plasma. Comparison with serum determination. Clin. Chem. Lab. Med. 41, 948–949. 10.1515/CCLM.2003.144 12940523

[B22] Fitts S. S. Guthrie M. R. (1995). Six-minute walk by people with chronic renal failure. Assessment of effort by perceived exertion. Am. J. Phys. Med. Rehabil. 74, 54–58. 10.1097/00002060-199501000-00009 7873114

[B23] Frih B. Mkacher W. Jaafar H. Frih A. Ben Salah Z. El May M.. (2017). Specific balance training included in an endurance-resistance exercise program improves postural balance in elderly patients undergoing haemodialysis. Disabil. Rehabil. [Epub ahead of print]. 10.1080/09638288.2016.1276971 28084833

[B24] Guigoz Y. Vellas B. Garry P. J. (1996). Assessing the nutritional status of the elderly: the mini nutritional assessment as part of the geriatric evaluation. Nutr. Rev. 54(1 Pt 2), S59–S65. 10.1111/j.1753-4887.1996.tb03793.x 8919685

[B25] Headley S. Germain M. Mailloux P. Mulhern J. Ashworth B. Burris J.. (2002). Resistance training improves strength and functional measures in patients with end-stage renal disease. Am. J. Kidney Dis. 40, 355–364. 10.1053/ajkd.2002.34520 12148109

[B26] Heinrich P. C. Castell J. V. Andus T. (1990). Interleukin-6 and the acute phase response. Biochem. J. 265, 621–636. 10.1042/bj2650621 1689567 PMC1133681

[B27] Heuck C. C. Ritz E. (1980). Hyperlipoproteinemia in renal insufficiency. Nephron 25, 1–7. 10.1159/000181745 6986036

[B28] Himmelfarb J. (2005). Hemodialysis complications. Am. J. Kidney Dis. 45, 1122–1131. 10.1053/j.ajkd.2005.02.031 15957144

[B29] Hopkins W. G. Marshall S. W. Batterham A. M. Hanin J. (2009). Progressive statistics for studies in sports medicine and exercise science. Med. Sci. Sports Exerc. 41, 3–13. 10.1249/MSS.0b013e31818cb278 19092709

[B30] Johansen K. L. Shubert T. Doyle J. Soher B. Sakkas G. K. Kent-Braun J. A. (2003). Muscle atrophy in patients receiving hemodialysis: effects on muscle strength, muscle quality, and physical function. Kidney Int. 63, 291–297. 10.1046/j.1523-1755.2003.00704.x 12472795

[B31] Li M. Tomlinson G. Naglie G. Cook W. L. Jassal S. V. (2008). Geriatric comorbidities, such as falls, confer an independent mortality risk to elderly dialysis patients. Nephrol. Dial. Transplant. 23, 1396–1400. 10.1093/ndt/gfm778 18057068

[B32] Lowrie E. G. Curtin R. B. LePain N. Schatell D. (2003). Medical outcomes study short form-36: a consistent and powerful predictor of morbidity and mortality in dialysis patients. Am. J. Kidney Dis. 41, 1286–1292. 10.1016/S0272-6386(03)00361-5 12776282

[B33] McKercher C. Sanderson K. Jose M. D. (2013). Psychosocial factors in people with chronic kidney disease prior to renal replacement therapy. Nephrology (Carlton) 18, 585–591. 10.1111/nep.12138 23876102

[B34] Moore G. E. Parsons D. B. Stray-Gundersen J. Painter P. L. Brinker K. R. Mitchell J. H. (1993). Uremic myopathy limits aerobic capacity in hemodialysis patients. Am. J. Kidney Dis. 22, 277–287. 10.1016/S0272-6386(12)70319-0 8352254

[B35] Moussouli M. Vlachopoulos S. P. Kofotolis N. D. Theodorakis Y. Malliou P. Kellis E. (2014). Effects of stabilization exercises on health-related quality of life in women with chronic low back pain. J. Phys. Act. Health 11, 1295–1303. 10.1123/jpah.2012-0426 24184617

[B36] Okumiya K. Matsubayashi K. Nakamura T. Fujisawa M. Osaki Y. Doi Y.. (1998). The timed “up & go” test is a useful predictor of falls in community-dwelling older people. J. Am. Geriatr. Soc. 46, 928–930. 10.1111/j.1532-5415.1998.tb02737.x 9670889

[B37] Painter P. (1994). The importance of exercise training in rehabilitation of patients with end-stage renal disease. Am. J. Kidney Dis. 24(1 Suppl. 1), S2–S9; discussion: S31–S32. 8023835

[B38] Painter P. Carlson L. Carey S. Paul S. M. Myll J. (2000). Physical functioning and health-related quality-of-life changes with exercise training in hemodialysis patients. Am. J. Kidney Dis. 35, 482–492. 10.1016/S0272-6386(00)70202-2 10692275

[B39] Painter P. Moore G. Carlson L. Paul S. Myll J. Phillips W.. (2002). Effects of exercise training plus normalization of hematocrit on exercise capacity and health-related quality of life. Am. J. Kidney Dis. 39, 257–265. 10.1053/ajkd.2002.30544 11840365

[B40] Parsons T. L. Toffelmire E. B. King-VanVlack C. E. (2006). Exercise training during hemodialysis improves dialysis efficacy and physical performance. Arch. Phys. Med. Rehabil. 87, 680–687. 10.1016/j.apmr.2005.12.044 16635631

[B41] Pennell P. Leclercq B. Delahunty M. I. Walters B. A. (2006). The utility of non-HDL in managing dyslipidemia of stage 5 chronic kidney disease. Clin. Nephrol. 66, 336–347. 10.5414/CNP66336 17140163

[B42] Podsiadlo D. Richardson S. (1991). The timed “Up & Go”: a test of basic functional mobility for frail elderly persons. J. Am. Geriatr. Soc. 39, 142–148. 10.1111/j.1532-5415.1991.tb01616.x 1991946

[B43] Pupim L. B. Flakoll P. J. Levenhagen D. K. Ikizler T. A. (2004). Exercise augments the acute anabolic effects of intradialytic parenteral nutrition in chronic hemodialysis patients. Am. J. Physiol. Endocrinol. Metab. 286, E589–E597. 10.1152/ajpendo.00384.2003 14678952

[B44] Rafferty M. R. Schmidt P. N. Luo S. T. Li K. Marras C. Davis T. L.. (2017). Regular exercise, quality of life, and mobility in Parkinson's disease: a longitudinal analysis of national Parkinson foundation quality improvement initiative data. J. Parkinsons Dis. 7, 193–202. 10.3233/JPD-160912 27858719 PMC5482526

[B45] Reboredo Mde M. Henrique D. M. Faria Rde S. Chaoubah A. Bastos M. G. de Paula R. B. (2010). Exercise training during hemodialysis reduces blood pressure and increases physical functioning and quality of life. Artif. Organs 34, 586–593. 10.1111/j.1525-1594.2009.00929.x 20497161

[B46] Ritchie C. Trost S. G. Brown W. Armit C. (2005). Reliability and validity of physical fitness field tests for adults aged 55 to 70 years. J. Sci. Med. Sport 8, 61–70. 10.1016/S1440-2440(05)80025-8 15887902

[B47] Rossier A. Pruijm M. Hannane D. Burnier M. Teta D. (2012). Incidence, complications and risk factors for severe falls in patients on maintenance haemodialysis. Nephrol. Dial. Transplant. 27, 352–357. 10.1093/ndt/gfr326 21652549

[B48] Segura-Orti E. Martinez-Olmos F. J. (2011). Test-retest reliability and minimal detectable change scores for sit-to-stand-to-sit tests, the six-minute walk test, the one-leg heel-rise test, and handgrip strength in people undergoing hemodialysis. Phys. Ther. 91, 1244–1252. 10.2522/ptj.20100141 21719637

[B49] Sietsema K. E. Amato A. Adler S. G. Brass E. P. (2004). Exercise capacity as a predictor of survival among ambulatory patients with end-stage renal disease. Kidney Int. 65, 719–724. 10.1111/j.1523-1755.2004.00411.x 14717947

[B50] Singh M. A. (2002). Exercise comes of age: rationale and recommendations for a geriatric exercise prescription. J. Gerontol. A Biol. Sci. Med. Sci. 57, M262–M282. 10.1093/gerona/57.5.M262 11983720

[B51] Steffen T. M. Hacker T. A. Mollinger L. (2002). Age- and gender-related test performance in community-dwelling elderly people: six-minute walk test, berg balance scale, timed up & go test, and gait speeds. Phys. Ther. 82, 128–137. 10.1093/ptj/82.2.128 11856064

[B52] van Vilsteren M. C. de Greef M. H. Huisman R. M. (2005). The effects of a low-to-moderate intensity pre-conditioning exercise programme linked with exercise counselling for sedentary haemodialysis patients in The Netherlands: results of a randomized clinical trial. Nephrol. Dial. Transplant. 20, 141–146. 10.1093/ndt/gfh560 15522901

[B53] Vellas B. Guigoz Y. Garry P. J. Nourhashemi F. Bennahum D. Lauque S.. (1999). The Mini Nutritional Assessment (MNA) and its use in grading the nutritional state of elderly patients. Nutrition 15, 116–122. 10.1016/S0899-9007(98)00171-3 9990575

[B54] Verbunt J. A. Seelen H. A. Vlaeyen J. W. Bousema E. J. van der Heijden G. J. Heuts P. H.. (2005). Pain-related factors contributing to muscle inhibition in patients with chronic low back pain: an experimental investigation based on superimposed electrical stimulation. Clin. J. Pain 21, 232–240. 10.1097/00002508-200505000-00006 15818075

[B55] Ware J. E. Jr. (2000). SF-36 health survey update. Spine (Phila Pa 1976) 25, 3130–3139. 10.1097/00007632-200012150-00008 11124729

[B56] Ware J. E. Jr. Sherbourne C. D. (1992). The MOS 36-item short-form health survey (SF-36). I. Conceptual framework and item selection. Med. Care 30, 473–483. 10.1097/00005650-199206000-00002 1593914

[B57] Whitney S. L. Wrisley D. M. Marchetti G. F. Gee M. A. Redfern M. S. Furman J. M. (2005). Clinical measurement of sit-to-stand performance in people with balance disorders: validity of data for the Five-Times-Sit-to-Stand Test. Phys. Ther. 85, 1034–1045. 10.1007/BF03325265 16180952

[B58] Wilund K. R. Tomayko E. J. Wu P. T. Ryong Chung H. Vallurupalli S. Lakshminarayanan B.. (2010). Intradialytic exercise training reduces oxidative stress and epicardial fat: a pilot study. Nephrol. Dial. Transplant. 25, 2695–2701. 10.1093/ndt/gfq106 20190243

[B59] Zigmond A. S. Snaith R. P. (1983). The hospital anxiety and depression scale. Acta Psychiatr. Scand. 67, 361–370. 10.1111/j.1600-0447.1983.tb09716.x 6880820

